# Characterisation of urban aerosol size distribution by radiocarbon and PIXE analyses in a middle-European urban environment for source identification: a pilot study

**DOI:** 10.1007/s11356-024-34215-8

**Published:** 2024-07-11

**Authors:** Anikó Angyal, Zoltán Szoboszlai, István Major, Mihály Molnár, Tamás Varga, Zsófia Török, Enikő Papp, Furu Enikő, Maria Gini, Manousos Ioannis Manousakas, Zita Szikszai, Zsófia Kertész

**Affiliations:** 1 HUN-REN Institute for Nuclear Research (ATOMKI), P.O Box 51, Debrecen, 4001 Hungary; 2 International Radiocarbon AMS Competence and Training Center (INTERACT), HUN-REN Institute for Nuclear Research (ATOMKI), P.O Box 51, Debrecen, 4001 Hungary; 3 Institute of Nuclear & Radiological Science & Technology, NCSR “Demokritos”, Energy & Safety, 15310 Athens, Greece; 4 Laboratory of Atmospheric Chemistry, Paul Scherrer Institute, 5232 Villigen, Switzerland

**Keywords:** Size distribution, Radiocarbon concentration, Elemental concentration, Smog period, Vegetation period

## Abstract

This study, conducted in Debrecen, Hungary, aimed to analyse atmospheric particulate matter (APM or PM) through radiocarbon and PIXE analyses during the winter smog (23–25 January) and spring (15–18 May) seasons. The information presented in this pilot study aims to provide insight into the importance of utilising detailed characteristics of the mass size distributions of fossil carbon (f_f_) and contemporary carbon (f_C_) content. Additionally, it seeks to compare these characteristics with the size distributions of various elements to enable even more accurate PM source identification. In winter, APM concentrations were 86.27 μg/m^3^ (total), 17.07 μg/m^3^ (f_C_) and 10.4 μg/m^3^ (f_f_). In spring, these values changed to 29.5 μg/m^3^, 2.64 μg/m^3^ and 7.01 μg/m^3^, respectively. Notably, differences in mass size distribution patterns were observed between the two seasons, suggesting varied sources for contemporary carbon. Biomass burning emerged as a crucial source during the smog period, supported by similar MMAD (Mass Median Aerodynamic Diameter) values and a strong correlation (*r* = 0.95, *p* < 0.01) between potassium and f_C._ In spring, a significant change in the concentration and distribution of f_C_ occurred, with a broad, coarse mode and a less prominent accumulation mode. F_f_ was found to have similar distributions as PM, with nearly the same MMADs, during both periods. Finally, a comprehensive comparison of modal characteristics identified specific sources for the various components, including biomass burning, vehicle exhaust, coal and oil combustion, vehicle non-exhaust, road dust, tyre abrasion, mineral dust and biogenic emission. This study showcases how using radiocarbon and PIXE analysis in size distribution data can enhance our understanding of the sources of PM and their effects on different size fractions of PM.

**Supplementary Information:**

The online version contains supplementary material available at 10.1007/s11356-024-34215-8.

## Introduction

PM is a well-documented pollutant with adverse effects on human health, the environment and climate. Despite numerous published studies on PM effects, we still lack information about the specific components and mechanisms responsible for these impacts. The intricate effects of PM are linked to its chemical composition and size fraction, and while there are many studies that focus on either aspect of PM, studies that take both into account simultaneously are limited. A comprehensive exploration of the intricate interplay between PM composition, size and their diverse aerosol-related effects is essential for advancing our understanding of atmospheric processes and informing effective strategies for air quality management.

Ambient particle size distributions exhibit pronounced temporal and spatial variability, reflecting local and regional disparities in emission sources, transport and ambient chemical and physical processes (Gini et al. 2022). Despite the existence of various size classifications for ambient aerosols in the literature, the nucleation (Aitken) mode (particle size < 0.1 μm, commonly referred to as ultrafine) and accumulation modes (ranging between ∼ 0.1 and ∼ 2 μm) are generally collectively defined as “fine” particles (Seinfeld et al. 1998). In many instances, the accumulation mode is further subdivided into two sub-modes: the condensation mode (∼ 0.1–0.5 μm), resulting from primary particle emission and the growth of smaller particles through coagulation and gas condensation, and the droplet mode (∼0.5–2.0 μm), likely stemming from the aqueous-phase processing of condensation mode particles. Morbidity and mortality associated with air pollution are primarily linked to fine particulate matter (PM_2.5_, i.e., particulate matter with an aerodynamic diameter < 2.5 μm), as the small size of these particles enables them to penetrate deeply into the lungs (Xing et al. 2016). Coarse mode material (particle size > 2.5 μm) primarily originates from primary emissions and/or natural sources.

PM composition can be varied, mainly comprised of organic compounds, metals, ions and other constituents (Achilleos et al. 2017). One significant subset deserving of attention is carbonaceous aerosols, which encompass both organic carbon (OC) and elemental carbon (EC). The distinct properties of carbonaceous aerosols not only influence the overall composition of PM but also hold specific implications for atmospheric processes and environmental impacts. Organic carbon, originating from sources like biomass burning and vehicle emissions, contributes to secondary organic aerosol formation and influences air quality. Elemental carbon, often associated with combustion processes, has direct implications for radiative forcing. Recognising carbonaceous particles as emerging pollutants underscores the need for targeted policies and strategies within the EU to mitigate their sources, understand their atmospheric behaviour, and protect public health and the environment. Addressing carbonaceous aerosols as a priority in the EU’s air quality management efforts is considered crucial for achieving sustainable and healthy urban environments. In Europe, the carbonaceous fraction of PM_2.5_ usually ranges between 30 and 60% of the total mass (Rogge et al. 1993; Temesi et al. 2003; Dusek et al. 2017). They can be emitted as primary components or formed by secondary processes from their precursors, e.g. incomplete burning of biomass and fossil fuels (EC), combustion and bioaerosol emissions (OC). Identifying the relative contribution of the different emission sources is important for understanding the formation processes and implementing effective mitigation measures.

Even though numerous studies are focused on apportioning the sources of carbonaceous aerosols (mostly organic), the distinction between different sources (e.g. liquid and solid fuel combustion) is still very challenging. One very important tool that can assist in achieving more reliable results is radiocarbon analysis, which is a relatively underused method due to its very challenging implementation. This analytical technique measures the ratio of carbon isotopes, particularly the abundance of radioactive ^14^C, which is produced in the atmosphere and incorporated into living organisms. It enables the crucial distinction between carbonaceous particles derived from fossil fuel combustion, lacking measurable ^14^C, and those originating from contemporary, biogenic sources containing measurable ^14^C, which is essential for accurately attributing contributions from sources like vehicular emissions, industrial processes and biomass burning. Incorporating radiocarbon data into source apportionment models improves their accuracy by offering precise information about the isotopic composition of carbonaceous particles, aiding in identifying and quantifying different sources (Vlachou et al. 2018). It also contributes to the understanding of secondary organic aerosol formation by distinguishing between fossil and contemporary sources of carbon, shedding light on the atmospheric processes involved (Szidat et al. 2004). The isotopic signature of carbonaceous aerosols can be used as an indicative metric for the success of fossil fuel mitigation policies.

Radiocarbon analysis has been successfully used in the past as a standalone method or as a supplementary tool for the source apportionment of carbonaceous particles (Handa et al. 2010; Szidat et al. 2004; Vlachou et al. 2018; Zhang et al. 2012, 2010; Salma et al. 2017). By implementing this approach, it is possible to get information about particle formation, accumulation and deposition processes for biogenic and anthropogenic emission sources. Biogenic OC depends on the activity of plants to emit reactive volatile species like monoterpenes and atmospheric oxidants that promote secondary organic aerosol formation, while anthropogenic OC correlates with black carbon or elemental carbon (Szidat et al. 2004). Radiocarbon analysis has been used for the study of haze events in China. It has been found that during high pollution events, fossil and non-fossil OC and EC concentrations did not increase in the smaller-sized particles (aerodynamic diameter < 0.25 μm), but highly increased in the larger size ranges. This indicates that local primary emissions did not increase during such events, and secondary formation was the driving force behind the high concentrations of particulate matter in the region (Ni et al. 2022). So far, ^14^C measurements on ambient PM samples have been made only on bulk samples (e.g. PM_10_, PM_2.5_ or PM_1.5_) in Europe. A few locations around Europe where this approach has been used in the past include different environments, such as an industrial harbour (Bonvalot et al. 2019), an urban site (Titos et al. 2017), an urban and coastal environment (Dusek et al. 2017), an urban background site (Major et al. 2015, 2021) and urban, coastal and forest sites (Masalaite et al. 2020). Even though size-segregated radiocarbon analysis data could provide even more information on organic aerosols’ sources and formation pathways, this information is still scarce.

Debrecen, located in eastern Hungary, boasts a population of over 220,000 residents. The city is surrounded by agricultural areas and features a mixed-fire power plant and an airport. According to the Regional Environment Agency (GOHBCH 2020), the majority of threshold exceedances, ranging from 92 to 95%, occur during the autumn–winter heating season. This is attributed partially to adverse atmospheric dilution and partly to significant air pollution resulting from the burning of solid fuels by residents. A previous study focusing on PM_2.5_ identified biomass burning and traffic as the main sources during smog periods, contributing to a combined total of 70% (Angyal et al. 2021). Furthermore, an extensive study covering PM_2.5_, radio and stable carbon isotopes was conducted from December 2011 to July 2014. The findings indicated that wood and biomass fuel combustion prevailed over coal or oil during the heating season, while biogenic emissions and transport played a major role during the non-heating period (Major et al. 2021).

Following the results of previous studies, we investigate the size distribution characteristics of radiocarbon components and compare them with the size distribution of elements to better identify the sources of PM and gain a deeper understanding of fossil and non-fossil-related source emissions. Due to the high cost and limited availability of radiocarbon measurements, we selected two sampling campaigns for this purpose: one during a smog episode (extreme PM pollution) and the other during the vegetation (i.e. non-heating) season. This study serves as a pilot study, showcasing the advantages of using techniques such as PIXE and radiocarbon data together on size-segregated PM samples to achieve a better understanding of PM sources.

To the best of our knowledge, this marks the first comprehensive pilot study examining the elemental composition of size-segregated atmospheric aerosols, coupled with an analysis of their radiocarbon content. The concurrent evaluation of these parameters enhances our understanding of distinct aerosol emission sources and formation processes.

## Methodology

### Receptor site

**Fig. 1:**
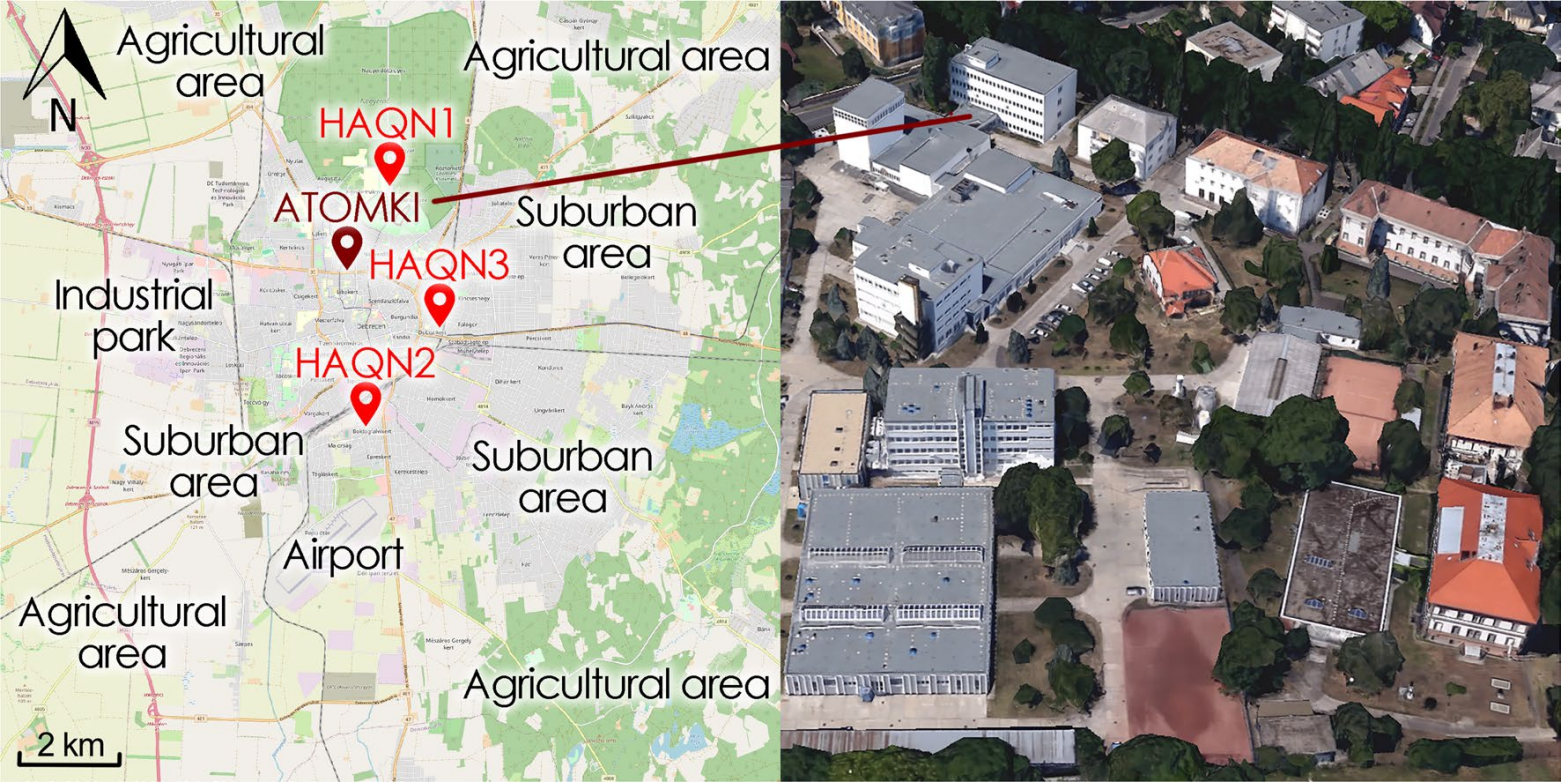
Left: the city of Debrecen with the sampling site (ATOMKI) and the locations of the monitoring stations (HAQN1, suburban background; HAQN2, urban background; HAQN3, traffic site). Right: expanded view of the sampling area

The measurement campaigns took place at the aerosol sampling station of ATOMKI in Debrecen, Hungary. The site is located at a height of about 5 m above street level on the roof of building VIII. The distance from the surrounding buildings is 4 m and approximately 20 m from vegetation. The map of Debrecen with the sampling site (ATOMKI) and the 3 official monitoring stations operated by the Hungarian Air Quality Network (HAQN) is shown in Fig. 1. Near or immediately surrounding the receptor site is a main road to the west, national road No. 33 to the south, and streets with tramways to the east and west. The sampling location can be considered an urban background site (Larssen 1999).

### Aerosol sampling

The haze event in January 2017 was selected because it represents the longest event recorded since 2011. On January 23, 2017, a smog alert was issued, prompting us to initiate the sampling campaign with the MOUDI impactor on that date to investigate the size distribution of PM. Haze events are infrequent and short-lived, making it impossible to conduct long sampling campaigns. To provide insight into the representativeness of the study events compared to other similar ones in the region, we have included size distribution data from three-day campaigns that took place in 2016 and 2018 in the supporting material (Figure S1, Supplementary Material). This inclusion demonstrates that the size distributions during haze events are comparable across different years. This consistency suggests that the results of this study are representative of typical conditions during such events. The sampling was carried out by a micro-orifice uniform deposit impactor nano-MOUDI II, model 122R, (MSP Corp.) (Marple et al. 2014) at a 30 L min^−1^ flow rate. The device separates the aerosol particles into 14-size fractions. The equivalent cut-off diameters at 50% efficiency of the impaction stages at a flow rate of 30 L min^−1^ are presented in Table 1.

**Table 1:** List of stages with the corresponding collected diameter range and cutting diameter

Stage	Diameter range (μm)	Cut-off point diameter (μm)
S1	> 18	18
S2	10–18	10
S3	5.6–10	5.6
S4	3.2–5.6	3.2
S5	1.8–3.2	1.8
S6	1–1.8	1
S7	0.56–1	0.56
S8	0.32–0.56	0.32
S9	0.18–0.32	0.18
S10	0.1–0.18	0.1
Spacer	0.056–0.1	0.056
11	0.032–0.056	0.032
12	0.018–0.032	0.018
13	0.01–0.018	0.01

**Fig. 2:**
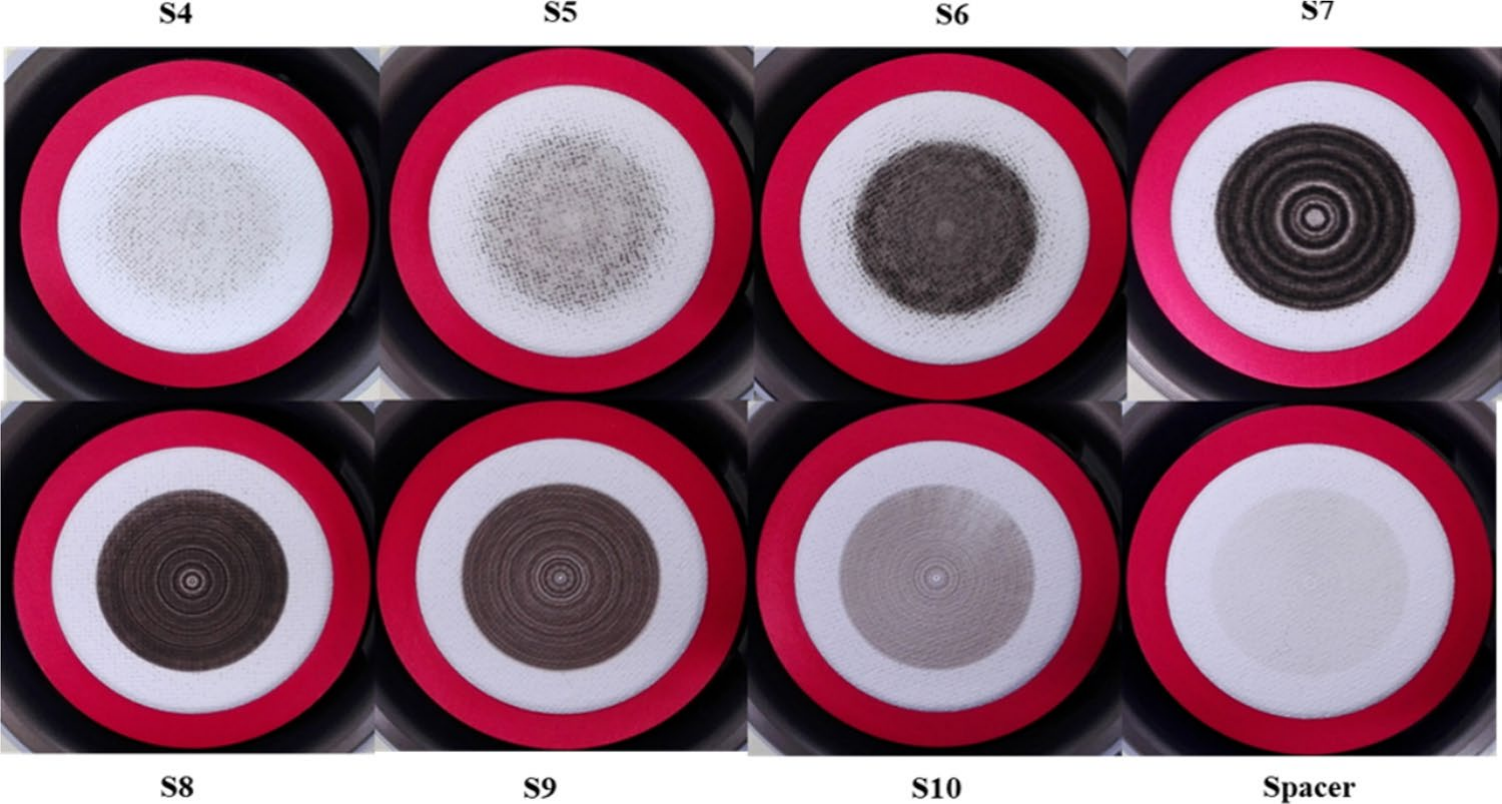
Examples of PM deposits on quartz-fibre filters collected by the nano-MOUDI-II impactor (stages from S4 to Spacer) in January 2017

Based on our former experiment, a 72-h sampling time was needed to collect a sufficient amount (at least 10 μg) of particulate carbon at each impactor stage to ensure proper analysis. Since the impactor stages continuously rotate during sampling (except for the last three nano-stages), PM deposits are circular and concentric (Fig. 2). Quartz-fibre filters were used as collection substrates (47-mm diameter for stages 1–11 and 90-mm diameter for stages 12–14). Before sampling, the filters were baked for at least 12 h in a muffle furnace oven at 850 °C to remove all carbonaceous contamination. The mass of the samples was determined by gravimetric analysis using a microbalance (Sartorius LE26P; accuracy 1 μg). The filters were conditioned at 26 °C and 36% relative humidity in a conditioning room for at least 24 h before weighting. A big segment of a sample (7/8) was used for the C-14 analysis, and PIXE measurements were performed on the tiny remnants of the filters. Aerosol samples were collected between 23–25 January and 15–18 May 2017. The meteorological conditions of the periods studied are described in detail later in the text.

### Chemical analysis

The elemental composition (for atomic number Z ≥ 6) of the aerosol samples was determined by PIXE analysis (particle-induced X-ray emission) (Maenhaut and Malmqvist 2002). The measurements were performed at the macro-PIXE chamber installed on the left 45° beamline of the 5 MV Van de Graaff accelerator of ATOMKI (Borbély-Kiss et al. 1985). A homogenous beam of 2 MeV protons was used for the irradiation. The beam spot had a diameter of 5 mm. The beam intensity was typically 20 nA, and the accumulated charge on each sample was 40 μC. A Canberra-type Si(Li) X-ray detector with a 30 mm^2^ active area and a 20 μm Be window was used, which was placed in 135° geometry to the incident beam. During the PIXE analysis, an additional 14.1 μm thick Al-absorbent was applied besides the usual 24 μm mylar absorber in order to get rid of the high Si X-ray background of the quartz filters. Although quartz substrate is not ideal for PIXE (or XRF) analysis, the determination of the elemental composition of the deposited aerosol layer is possible and is done in other laboratories too (Žitnik et al. 2005; Chiari et al. 2015). The analysis was performed on all size fractions. However, in the case of the nanosize fractions that were collected on the filters with a 90-mm diameter, the elemental concentrations were under or close to the detection limits. In addition, the mass measurement of these filters was also inadequate due to their size; therefore, these results are not presented in this paper. A blank correction was performed for each filter. The obtained X-ray spectra were evaluated with the GUPIXWIN software package (Campbell et al. 2010). The accuracy of the elemental analysis was checked by analysing National Institute of Standards and Technology (NIST) standard reference materials SRM610 (trace elements in glass) and SRM 2783 (APM on filter media). This latter aerosol standard represents a PM_2.5_ material with elemental concentrations typical of those of an urban industrial area.

For the C-14 analyses, samples were prepared using the sealed tube combustion technique, which was designed for offline combustion and CO_2_ purification of aerosol samples at the ICER Laboratory (Janovics et al. 2018). Sample combustion was performed within sealed test tubes (ø9 mm, 120 mm) made of borosilicate glass. Test tubes were loaded with the aerosol filters (3/4 section from each) and were evacuated to vacuum (< 10^−4^ mbar) before being sealed with a gas torch. Beside the sample, ~ 15 mg MnO_2_ and ~ 5 mg Ag wool reagents were also placed into the tubes in order to oxidise and catalytically clean the carbonaceous material (at 550 °C for 3 days). A dedicated vacuum line with water- and CO_2_ traps and a calibrated known-volume reservoir with a pressure gauge were applied to cryogenically extract and clean the produced CO_2_ gas. The quantity of CO_2_ was determined by high-precision pressure measurement, and finally, the yield was calculated based on the initial and final masses of the samples. The amount of the generated CO_2_ gas and, thus, the carbon content can be calculated from the ideal gas law. This carbon amount produced by the combustion of the filter sections was used to calculate the total carbon content (TC) of the aerosol samples (Major et al. 2018).

The radiocarbon measurement of the small aerosol samples (10–300 μg C in CO_2_) was performed on the EnviroMICADAS accelerator mass spectrometer (AMS) of HEKAL Laboratory using its dedicated gas ion source interface (GIS). Purified CO_2_ samples from aerosols were cryogenically transferred and sealed into glass tubes (outside diameter (OD) = 4 mm and 60 mm long) under vacuum. Those small glass ampoules are handled by GIS connected directly to the C-14 analyzer AMS system (Molnár et al. 2021).

Besides the actual aerosol samples, several sample blanks using fossil CO_2_ gas blank samples (Linde, Répcelak, Hungary) were also prepared under the same conditions as the aerosol samples (including the combustion step by MnO_2_) and measured for ^14^C to qualify the preparation. Based on the measurement of background samples, 1.0 ± 0.1 μg of modern carbon contamination per prepared filter section was used in the blank correction. Normalisation of the ^14^C measurements was performed by measuring similar sized (50–100 μg C) gas targets made from NIST SRM 4990C oxalic acid II C-14 standards. The average relative 1 sigma error achieved this way for modern samples was ± 1.0 rel. %. The ^14^C/^12^C ratios of the samples are given in units of the internationally used f_M_ (fraction Modern) value (Burr et al. 2009). MICADAS AMS measures the ^13^C/^12^C ratio for isotope fractionation correction online during ^14^C analyses, and it is used in the data correction and reduction process (Wacker et al. 2010).

In the case of fossil fuels, all of their total ^14^C content has already decayed during their very long geological storage; therefore, their f_M_ value is 0. However, in biological aerosols, which are formed by recent biological sources, f_M_ values are the same as for the atmosphere and the current biological materials (f_M_ ~ 1). As the majority of the currently combusted firewood was growing during the period of the ^14^C bomb peak due to the nuclear weapon tests (between 1960 and 2000) (Currie et al. 1989), the aerosol particles containing carbon from wood combustion have a radiocarbon activity slightly higher than that of the present atmosphere. The ^14^C level of the materials from the combustion of this bomb-effected wood is on average 1.08 times higher than that before the bomb-effect (Szidat et al. 2006, 2009).

Therefore, the fraction of f_C_ in the atmospheric aerosol samples can be calculated using the following formula (Heal et al. 2011):

1 $${f_C}={f_M}/1.08$$

The fraction of f_f_ is the remaining fraction of the total carbon since the main sources of TC in aerosols are recent biological emissions, wood/biomass burning, fossil fuel burning and atmospheric oxidation of VOCs (volatile organic compounds):

2 $${f_f}=1-{f_C}$$

By definition, f_C_ is between 0 and 1, where 0 means that the entire aerosol is fossil-derived without any recent biological carbon content, and 1 means that the whole carbon content is recent.

### Data analysis

#### Enrichment factor calculation

In order to have an indication of the types of sources of PM, the crustal enrichment factor (EF) of each element was calculated according to the following equation:

3 $$\text{EF }=\frac{\frac{{X}_{PM}}{{Si}_{PM}}}{\frac{{X}_{Crust}}{{Si}_{Crust}}}$$

where X is the concentration of the element of interest and Si is the concentration of a reference element, which is predominately of natural origin and whose mass is conserved. By convention, an EF < 10 is indicative of the elements’ crustal origin, due to some uncertainty related to the natural variation of crustal composition, while elements with an EF > 10 are attributed as having originated from anthropogenic sources. The EFs were calculated on the basis of the elemental concentrations in Earth’s upper crust (Mason 1952).

#### Multi-modal analysis of mass size distributions

The size-fractionated mass size distributions obtained with the MOUDI cascade impactor were inverted into smooth size distributions using the MICRON inversion algorithm (Wolfenbarger and Seinfeld 1990). The inversion algorithm takes into account the collection characteristics of each impaction stage, as reflected by the impactor collection efficiency curves, while assuming that each mass size distribution can be reproduced by the sum of unimodal log-normal distributions. Then, the inverted size distributions were fitted by the sum of up to six log-normal distributions, employing the minimum Root Mean Square Error (RMSE%) to assess the goodness of fit. Each mode was characterised by the MMAD (characteristic parameter to define the mean size of aerosol particles for each size mode), the geometric standard deviation (GSD) and the mass concentration. The fitting methodology applied in the present study has already been described in previous studies (Hussain et al. 2011; Zwozdziak et al. 2017). In total, 14 datasets (mass size distributions) were analysed, which correspond to the mass size distributions of ambient particulate matter and its specific chemical components (i.e. major and trace elements, contemporary and fossil-derived carbon).

## Results and discussion

### PM_2.5_, PM_10_, CO and NO_x_ concentrations

**Fig. 3:**
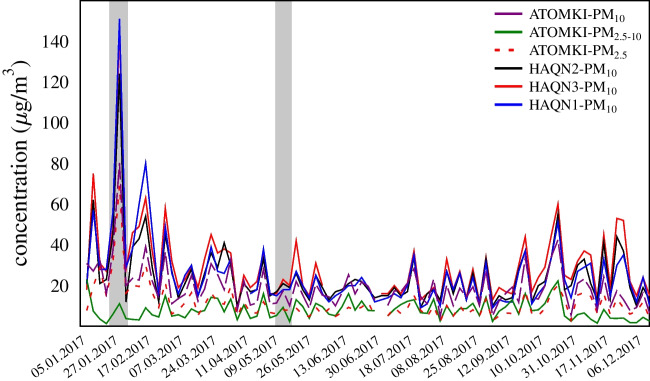
Daily (24 h) PM_10_, PM_2.5_ and PM_2.5–10_ mass concentrations measured at ATOMKI and PM_10_ concentrations measured at the Hungarian Air Quality Network stations in 2017. The grey bars indicate the investigated periods

To accurately depict the air quality of the city during the campaigns, concentration levels of all air pollutants, such as PM_10_, CO and NOx, served as valuable indicators. Since only PM is monitored at our institute, the CO and NO_x_ levels were taken from official monitoring station (HAQN) data. Firstly, the PM_10_ concentrations measured at ATOMKI were compared with the PM_10_ concentrations measured at the 3 HAQN stations in Debrecen in 2017, and the time series are presented in Fig. 3. PM_2.5_, PM_10_ and PM_coarse_ have been monitored regularly at the ATOMKI station since 1998 by collecting 24-h PM_2.5_ and PM_2.5–10_ samples two times a week (Furu et al. 2015; Kertész et al. 2024).

In general, PM_10_ concentrations measured at ATOMKI were lower than those reported by the HAQN stations. The differences between automatic (as in HAQN stations) and standard offline PM mass concentration measurements were discussed in our previous study (Angyal et al. 2021). Automated measurements are based on the β-ray absorption method (BAM), which has been shown to be affected by water uptake in conditions of high relative humidity (Shin et al. 2011), with greater deviations occurring at higher ambient RH (Chang and Tsai 2003). Therefore, it is necessary to perform a comparison between offline and online PM mass concentration methods for validation. In 2017, PM_10_ concentrations at ATOMKI correlated well with the PM_10_ (*r* = 0.76, p < 0.01) values of HAQN1 and HAQN2 (r = 0.72, p < 0.01) (Table S1, Supplementary Material). Therefore, for this study, the hourly data from the monitoring station HAQN1 were utilised for the sampling periods, as this suburban background station is closest (~ 900 m) to the sampling point and has the same characteristics.

**Fig. 4:**
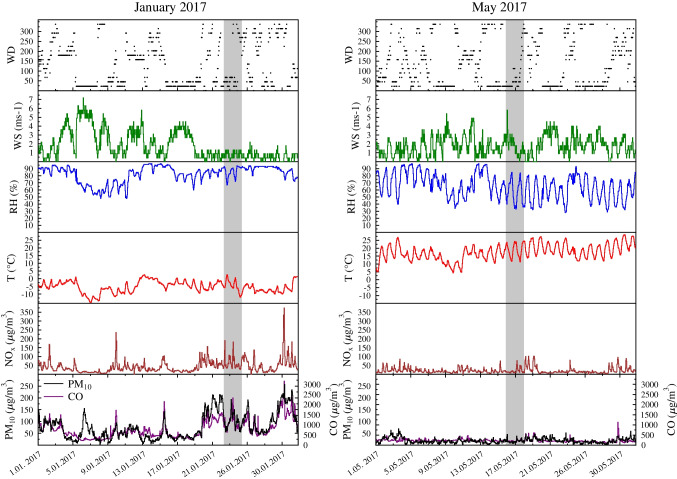
Temporal variation of meteorological parameters (T, RH, P, WS, WD), PM_10_, CO and NO_x_ in January (smog period) and in May 2017 (vegetation period)

Meteorological parameters, PM_10_, CO and NO_x_ levels recorded at HAQN1 during January and May 2017 are presented in Fig. 4. The average PM_10_ concentration during the investigated smog period at HAQN1 was 103.1 ± 29.5 μgm^−3^ with a maximum of 194.0 μgm^−3^ on January 24, 2 a.m., associated with north-east (NE) wind direction.

During the sampling period from 15 to 18 May, the average PM_10_ concentration was 16.8 ± 7.4 μgm^−3^.

In this work, the APM pollution is described in two periods, which can be characterised by different meteorological and air quality conditions. Concentration levels of the main air pollutants were observed to be 3–7 times higher during the smog period (sp) compared to the vegetation period (vp), with the most significant difference—a factor of 6.5—seen in the concentration of particulate matter. Concerning the different air pollutants, a moderate correlation was observed between PM_10_ and NO_x_ in both sampling campaigns, while PM_10_ was strongly and moderately correlated with CO during winter and spring, respectively (Table S2, Supplemetary Material).

## Mass size distributions of PM, fossil and contemporary carbon

The mass concentration (summarised for size fractions from 0.1 μm to > 18 μm) measured at ATOMKI was 86.3 ± 29 μgm^−3^ during the 72-h-long campaign in the smog and 29.5 ± 2.6 μgm^−3^ in the vegetation period. The mass concentration of the total PM for the smog period was almost three times as high as in the vegetation period. The total contemporary carbon concentration was 17.1 ± 6.3 μgm^−3^ during smog and was markedly lower (2.6 ± 0.4 μgm^−3^) in May. In contrast to this, the values of the total fossil-derived carbon concentration were quite similar (for winter: 10.4 ± 1.5 μgm^−3^, for spring: 7 ± 0.3 μgm^−3^) in both campaigns.

**Fig. 5:**
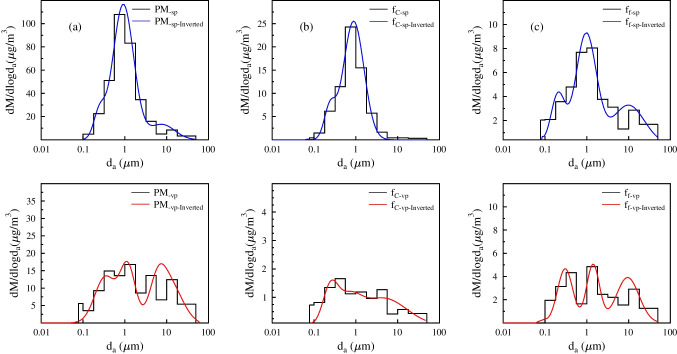
Mass size distributions of PM (**a**), f_C_ (**b**) and f_f_ (**c**) in aerosols from an urban background site in Debrecen, Hungary, during a smog and a vegetation period in 2017

The size distribution patterns of PM mass, f_f_ and f_C_ from both sampling campaigns are shown in Fig. 5. As mentioned earlier, the ultrafine fraction of all components could not be measured; therefore, only the modes with MMAD > 0.1 μm were investigated. The accumulation mode could be divided into two sub-modes: condensation and droplet modes. The MMAD of f_C_ in the condensation mode was 0.24 and 0.25 μm, and for f_f_, it was 0.2 and 0.3 μm, whereas the MMAD of the droplet mode was 0.85 and 0.67 μm for f_C_ and 0.96 and 1.41 μm for f_f_ during the winter and spring campaigns, respectively. A major fraction of the aerosol mass (~ 80%) was found in the droplet mode in the smog period, likely formed from aqueous-phase processing of condensation mode particles. The stable weather conditions and especially the high relative humidity (average RH: ~ 86%) facilitated the growth of condensation mode particles, leading to the formation of droplets. This process was probably followed by the dissolution of some atmospheric gases into droplets and by aqueous-phase chemical reactions. The phenomenon is well described in aerosol studies, and it was observed earlier for inorganic ions (e.g. SO_4_^2−^) (Hering 1982; Hering et al. 1997; Plaza et al. 2011; Wang et al. 2021) and elemental components (Salma et al. 2005) under high relative humidity conditions.

The two main modes (accumulation, from 0.1 to ∼ 2 μm, and coarse, above 2 μm) are observed in both campaigns for all main components (Fig. 5).

As shown in Fig. 5, as well as in Table 2, the mass size distribution of carbon components follows that of PM, with nearly the same MMADs in condensation and droplet mode during the winter campaign. The droplet mode was the major contributor to PM mass, f_C_ and f_f,_ with MMADs of 0.92, 0.85 and 0.96 μm, respectively (Table 2). The similar size distributions of the measured species (PM mass, f_C_, f_f_) indicate that they were internally mixed within this mode. Together, these suggest a common evolution and ageing mechanism.

**Table 2:** Modal characteristics of PM, f_C_ and f_f_ mass size distributions for the smog and vegetation period in 2017. GSD - geometric standard deviation, MMAD - mass median aerodynamic diameter

		Accumulation mode												
		Condensation mode			Droplet mode			Coarse mode						Total
		GSD	MMAD	Mass conc	GSD	MMAD	Mass conc	GSD	MMAD	Mass conc	GSD	MMAD	Mass conc	Mass conc. (μg/m^3^)
			(μm)	(μg/m^3^)		(μm)	(μg/m^3^)		(μm)	(μg/m^3^)		(μm)	(μg/m^3^)	
Smog period	PM	1.36	0.24	7.2	1.77	0.92	69.31				2.03	7.68	9.76	86.27
	f_C_	1.36	0.24	2.05	1.76	0.85	14.56				1.69	6.58	0.46	17.07
	f_f_	1.46	0.2	1.71	1.76	0.96	5.67				2.41	9.89	3.02	10.40
Vegetation period	PM	1.78	0.34	8.54	1.52	1.18	7.54				2.08	8.50	13.4	29.5
	f_C_	1.51	0.25	0.58	1.9	0.67	0.59	3.64	4.49	1.47				2.64
	f_f_	1.72	0.3	2.48	1.43	1.41	1.92				1.87	9.49	2.61	7.01

During the vegetation period, the droplet mode accounted for 26% of the total PM mass, while the coarse mode reached 45%. As for the carbon components, the concentrations did not change strongly in the smallest size range (aerodynamic diameter < 0.35 μm), while they decreased significantly in the droplet mode. Furthermore, the distribution patterns of the two carbon components are quite different in spring, which can be attributed to the different formation pathways.

**Fig. 6:**
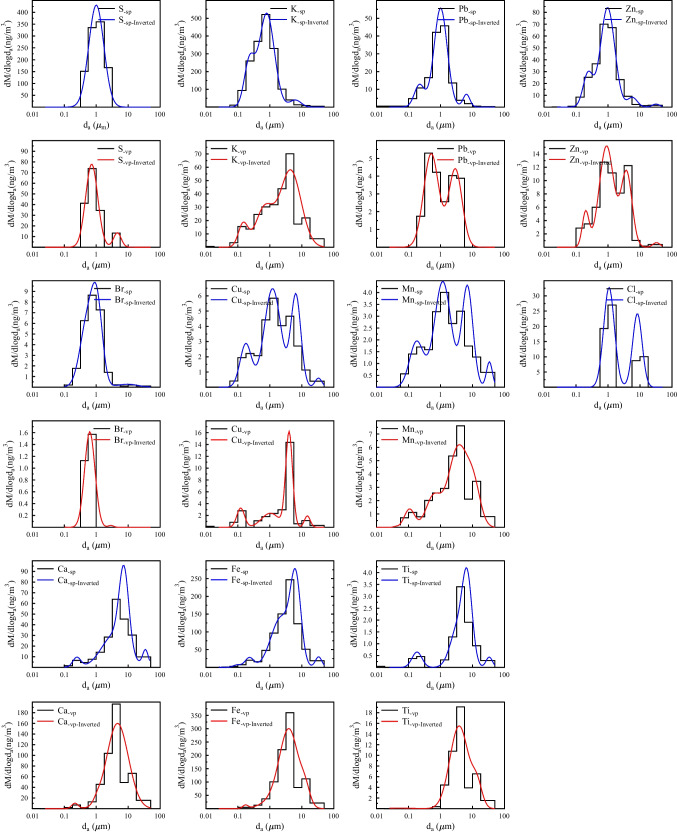
Mass size distributions of elemental composition in aerosols from an urban background site in Debrecen, Hungary, during the smog and vegetation period, 2017

Among the measured constituents, contemporary carbon showed the highest mass contribution (20%) to the PM mass during smog. The modal characteristics of the contemporary carbon mass size distributions were similar to the ones for the PM mass size distributions in the condensation and droplet size ranges (Table 1). This behaviour underlines the fact that a major part of the aerosol mass was associated with contemporary carbon sources. According to the size distribution patterns and the fitted MMADs (Table 2), potassium exhibited the greatest similarity with contemporary carbon (Fig. 6). The MMAD values of the two constituents were identical for both the condensation mode (f_C_: 0.24, K: 0.23) and the droplet mode (f_C_: 0.85, K: 0.80 μm). This similarity and the strong correlation (r = 0.95, *p* < 0.01) between these two components throughout the size ranges indicate unambiguously that these components had common sources, most probably biomass burning (BB) from residential heating. This is not an unexpected result since both constituents are markers of BB (Andreae et al. 1998). Aerosol studies dealing with BB have presented both unimodal and bimodal size distributions with patterns depending on several factors (e.g. fuel type, combustion environment, burning conditions, measurement technologies and atmospheric ageing) (Chen et al. 2017a). The presence of condensation mode could be attributed to the freshly emitted wood burning particles. This is supported by other aerosol studies in which freshly emitted wood smoke particles were observed in the 0.05–0.2 μm size range (Li et al. 2007; Chen et al. 2017b). Concerning wood burning, the Aitken mode was also detected by Bernardoni et al. (2017). They found a very similar MMAD value for the condensation mode (0.21 μm) compared to the results of this study.

Several papers presented BB-related size distributions (Hays et al. 2005; Reid et al. 2005; Lin et al. 2010; Salma et al. 2013). In a previous study conducted in Southern China, the highly resolved mass size distributions of HULIS-C (carbon in humic-like substances) were presented (Lin et al. 2010). It is known that BB emits a large amount of HULIS (Salma et al. 2010); thus, it would be relevant to compare it with the mass size distributions reported here. The structure of the HULIS-C size distribution and the corresponding MMADs showed good agreement with our findings in the case of contemporary carbon. Those distributions also consisted of condensation, droplet and coarse modes with MMADs in the size ranges of 0.23–0.28 μm, 0.63–0.87 μm and 4.0–5.7 μm, respectively. In our study, the droplet mode represents 85% of the total contemporary carbon, while the condensation mode is 12% during the smog period. These results are close to those reported by Lin et al. (2010); they found that the droplet mode dominated the concentration of HULIS (81%), and the condensation mode accounted for 12%.

For the vegetation period, the structure of the distribution of contemporary carbon was quite different from both PM and f_f_ mass size distributions. Although three modes were resolved, they appeared much broader and, therefore, merged, with smaller diameters and larger GSD, especially for the coarse and droplet modes, differing from those of f_f_ and PM for this campaign (Table 2).

The mass size distribution of the fossil fraction was the most complicated, reflecting the impact of multiple fossil carbon emission sources. The structure of the size distribution also indicated condensation, a droplet and a coarse mode during both campaigns. In an urban area, fossil carbon derives from several sources: diesel fuel, gasoline fuel, lubricating oils, oil and coal combustion, etc. (Sanderson et al. 2014). For these reasons, the size distribution of f_f_ is discussed together with the elemental distributions to better identify sources.

### Possible sources based on the mass size distribution of the elemental components

The size distribution patterns of the elemental components for each campaign are shown in Fig. 6; their modal characteristics can be found in Table 3. Among the measured elemental components, potassium presented the highest concentration during the smog period. To assess the impact of anthropogenic emissions on the observed concentrations of elements, enrichment factors (EFs) were estimated for summarised size fractions. EFs ranged from 1 to 10 for mineral dust elements such as Ca, Ti, Mn and Fe, indicating their natural origin from soil resuspension (Salma et al. 2001). Furthermore, based on their estimated EF values (> 10), the elements generally associated with anthropogenic emissions (S, Cu, Zn and Pb) were predominantly found in the fine fraction during both campaigns. However, their concentrations were six times higher in the smog period. For potassium, EF shows that anthropogenic emissions dominated during the smog period, while natural emissions dominated during the warmer period.

**Table 3:** Modal characteristics of elemental composition for the investigated smog and vegetation period in 2017. GSD - geometric standard deviation, MMAD - mass median aerodynamic diameter

		Accumulation mode																
		Condensation			Droplet			Coarse									Total	
		GSD	MMAD	Mass conc	GSD	MMAD	Mass conc	GSD	MMAD	Mass conc	GSD	MMAD	Mass conc	GSD	MMAD	Mass conc	Mass conc	EF
			(μm)	(ng/m^3^)		(μm)	(ng/m^3^)		(μm)	(ng/m^3^)		(μm)	(ng/m^3^)		(μm)	(ng/m^3^)	(ng/m^3^)	
Smog period	S				1.72	1	255										255	1569
	Cl				1.47	1.08	13.8				1.41	8.27	9.03				23	281
	K	1.45	0.23	105	1.73	0.8	320				1.5	6.26	17.9				443	27
	Ca	1.33	0.25	2.91				2.51	3.69	30.44	1.41	7.36	27.2	1.23	34.75	3.43	64	3
	Ti	1.43	0.18	0.26				1.69	3.45	0.98	1.43	6.89	1.4	1.26	33.37	0.11	3	1
	Mn	1.65	0.18	1.05	1.82	1.18	2.92				1.49	6.93	1.84	1.24	33.83	0.25	6	10
	Fe	1.37	0.23	9.55				2.09	2.27	116.49	1.44	6.45	92.2	1.26	33.37	7.74	226	7
	Cu	1.51	0.18	1.31	1.82	1.21	4.33				1.43	6.5	2.41	1.27	33	0.16	8	239
	Zn	1.43	0.24	11.1	1.67	0.97	47.5				1.41	5.79	3.08	1.3	31.54	0.75	62	1427
	Br				1.81	0.8	6.39				2.11	9.6	0.23				7	
	Pb	1.44	0.22	5.08	1.6	1.01	29.2				1.28	6.48	1.97				36	4462
Vegetation period	S				1.55	0.72	37	1.32	4.57	4.06							41	54
	K	1.41	0.15	6.43	1.89	0.69	20.3	2.12	4.43	47.2				1.54	20.72	1.25	75	1
	Ca	1.26	0.22	2.61	1.27	1.06	1.65	2.18	4.63	136							140	1
	Ti							1.95	3.84	11.3				1.43	13.96	1.6	13	1
	Mn	1.44	0.11	0.55	1.7	0.6	1.3	2.05	3.66	4.73				1.59	11.41	1.17	8	3
	Fe	1.21	0.17	2.65	2.54	1.69	38.3	1.96	4.04	202				1.4	12.84	20	263	2
	Cu	1.32	0.12	0.98	1.88	1.04	1.64	1.28	3.98	4.27				1.23	14.81	0.44	7	45
	Zn	1.27	0.2	1.38	1.69	0.9	8.67	1.48	3.8	4.79				1.29	32.67	0.19	15	73
	Br				1.45	0.63	0.66										1	
	Pb				1.72	0.51	3.12	1.62	2.91	2.3							5	142

Concerning the mass size distributions, several elements exhibited similarities with each other and with the carbonaceous components, suggesting identical sources or similar formation pathways. In general, the peak in the fine mode is attributed to a combination of different combustion sources (transport engine emissions, biomass burning, coal combustion, etc.), some of which are more obvious in winter, while the peak in the coarse mode is mainly due to road dust or soil.

In the following paragraphs, the three size modes—condensation, droplet and coarse—are discussed separately.

(1)  *Condensation mode*: Most of the detected elements (K, Ca, Mn, Fe, Cu, Zn and Pb) and carbon components (f_C_ and f_f_) showed a small peak in the condensation mode (Fig. 6) during smog, suggesting that these were components of freshly emitted aerosol particles and probably originated from gas-to-particles transformation or combustion processes. The MMAD values of these components were approximately 0.20 μm, indicating that they originate from high-temperature processes. In the case of Fe and Ca, there was a small mode with MMAD of ~ 0.23 μm, which supports our assumption that this peak was associated with vehicle emissions. Using a MOUDI impactor, Schauer et al. (2006) also observed similar submicron peaks in the Fe and Ca mass size distributions in a roadway tunnel, which were attributed to tailpipe emissions of lubricating oils. The MMAD of Mn and Cu in the condensation mode was 0.18 μm, which can be attributed to the effect of the evaporation of brake materials. In addition, Pb and Zn showed similar MMAD at condensation (Zn: 0.24 and Pb: 0.22 μm), suggesting that they are from a common source. According to the size distribution patterns and the fitted MMADs (Table 2), potassium exhibited the greatest similarity with contemporary carbon (Fig. 6).

During the vegetation period, PM and f_f_ showed concentrations similar to those in the condensation mode as in the smog period. For the other components (f_C_, K, Ca, Mn, Fe, Cu and Zn), only slight peaks were observed, while concentrations of some elements (e.g. S, Ti, Br and Pb) were below the detection limit. This is due to the lower emissions from high-temperature processes, in particular the cessation of emissions from various combustion processes, which are enhanced by the accelerated condensation of semi-volatile organic compounds. For example, K and f_C_ did not show the same MMAD values, confirming that biomass combustion, one of the high-temperature processes, was not observed during this time. However, the concentration of f_f_ was higher than in the cold period. The MMAD values for PM and f_f_ were similar (PM: 0.34, f_f_: 0.30), indicating that traffic was still mainly responsible for this peak.

(2) *Droplet mode*: The majority of the mass of most elemental components (S, Cl, K, Mn, Cu, Zn, Br and Pb) and carbon components (f_C_ and f_f_) were found in the droplet mode (MMADs ~ 1 μm) during the smog campaign. The MMAD values of most elements (S, Cl, Br, Mn, Cu, Zn and Pb) were similar to that of fossil-derived carbon (MMAD = 0.96 μm), suggesting that these elements were derived from fossil sources. Sulphur is usually present in atmospheric aerosols in the form of sulphates, and they are almost solely of secondary origin (Viana et al. 2008). The mass size distribution of sulphates usually contains the three modes (Plaza et al. 2011): ultrafine, accumulation and coarse mode. Most of the condensation mode sulphate arises from gas-phase photochemical oxidation of SO_2_, followed by gas-to-particles transformation (Seinfeld et al. 1998), while droplet mode sulphate could be formed through aqueous oxidation of SO_2_ in fog/cloud droplets (Meng and Seinfeld 1994). Since sulphate (as a secondary aerosol) can mix with primary and secondary particles, discriminating between the possible sources is a challenge. The sulphur mass was concentrated in the droplet mode, which can be explained by the growth of condensation mode particles through water accretion.

The modal structures of lead and zinc were very similar during the smog period. Both elements had similar MMADs at the droplet mode (Zn: 0.97 and Pb: 1.01). A peak with a similar MMAD in the droplet mode was also present in the size distribution of sulphur and fossil-derived carbon. This suggests that these components were mixed in the drop-phase reactions. Zn and Pb are typical compounds of vehicle emissions and coal combustion (Viana et al. 2008; Ondráček et al. 2011; Duan and Tan 2013; Bernardoni et al. 2017). In the case of vehicle emissions, their source can be lubricating oil additives and fuel additives (Sanderson et al. 2014). Both sources are of fossil origin, which explains the similar MMADs of Zn, Pb and fossil carbon in the droplet mode. It is likely that aerosol particles from both coal combustion and vehicle emission sources are mixed; therefore, the presence of other signature elements can distinguish between these two sources. The typical traffic-related fossil fuels are diesel and gasoline. In the literature, only a limited number of data are available concerning the tracers of diesel and gasoline engine exhaust. Values of Zn/Pb between 0.8 and 1.8 indicate gasoline and diesel emissions (Arditsoglou and Samara 2005); values around 1.2 represent combustion of oil (Herut et al. 2001) and values around 1.8 have been reported for municipal incinerator emissions (Polissar et al. 2001). In our case, the Zn/Pb ratio was 1.6 during the smog period in the droplet mode and is in the range of 0.8–1.8, which still suggests a mixed origin of the tracers.

Cu and Mn are also typical traffic-related elements in the aerosol literature (Viana et al. 2008; Sanderson et al. 2014). In the urban environment, both elements could be associated with exhaust and with non-exhaust traffic sources. Nevertheless, fly ash particles from coal burning can also contain a minor amount of Mn (Zhang et al. 2017). In the case of exhaust sources, both diesel and gasoline fuels could contain Cu and Mn (Sanderson et al. 2014). Lin et al. (2005) found that gasoline vehicles were stronger emitters of Cu and Mn than diesel vehicles due to the metallic additives. Using a three-way source apportionment method, Bernardoni et al. (2017) could identify two traffic factors in the accumulation mode: (1) factor (characterised by a high relative contribution of Zn) was associated with diesel vehicles, while the (2) factor (characterised by high contributions of Cu and Mn) was related to gasoline vehicles. In our case, the modal structure of these elements showed high similarities, and their elemental ratio remained constant (Mn/Cu = 0.67–0.80) in almost the whole size range, implying that they originated from the same source during the smog period. Since their size distribution differed from that of the fossil-derived carbon, it was inferred that their emission were not related to fossil sources. This was supported by the fact that the addition of Mn to gasoline was restricted in Hungary to 6 mg l^−1^ from June 2011 by Hungarian laws (Law LXXVII/2011, Decrees 30/2011); therefore, gasoline cannot be a significant source of ambient Mn in Hungary. Hence, Cu and Mn most probably originated from non-exhaust sources, namely brake abrasion. These elements from brake wear can originate either from abrasive processes or from the volatilisation of metals due to heating (Sanderson et al. 2014). Since these are different processes, it is likely that their products are in two separate modes.

Biomass burning is a potential source of accumulation mode Cl since KCl is a typical constituent of the BB smoke particles (Chen et al. 2017a), and BB emission was significant during the investigated period. However, the size distribution of K, f_C_ and Cl showed only a slight similarity, which means that Cl had additional important sources. For example, at that time, we detected several emission episodes in the PM_2.5_ fraction when Cl was associated with metals (such as Zn, Cu and Pb), the origin of which was presumably industry or waste incineration (Angyal et al. 2011). Nevertheless, Cl is a very reactive element; therefore, it is difficult to connect it to its proper emission source. Further investigation is needed to answer this problem.

In the vegetation period, the MMAD values and size distributions of most components (f_C_, S, K, Mn, Br and Pb) shifted in the droplet mode towards a smaller size, and the concentrations were quite lower. This can be attributed to higher temperatures (av 18 °C in spring) and the lower relative humidity (< 70%), which reduced heterogeneous aqueous reactions. As mentioned above, the lack of combustion of fuels such as coal, oil and biomass can be observed in warmer seasons. The size distributions of S remained similar to those detected during the smog period, while a shift was observed for Pb and Zn. The MMAD values of Cu and Zn were also similar, suggesting that vehicle emissions were also detectable during this campaign. This is confirmed by the fact that the concentration of fossil carbon did not decrease to the same ratio as the other components did in this mode. Besides, the MMAD values of PM and f_f_ shifted towards a larger size together with soil-derived elements such as Ca and Fe. It is assumed that the result of the resuspension of fossil carbon soot particles attached to dust is already present in the droplet mode. The MMAD values of biogenic emission markers (K and f_C_) were also identical for the droplet (f_C_: 0.67, K: 0.69), and their concentrations were much lower than during the smog period. Based on these results, it is assumed that the natural emission of biogenic-derived carbonaceous aerosols dominated during this period.

(3) *Coarse mode*: Ca, Fe and Ti appeared to have most of their mass in the coarse mode, pointing out the significant role of the local road and/or soil resuspension processes in their mass concentration levels. The similarity in size distribution patterns observed in this study indicates that probably common sources contribute to the PM mass concentrations for both sampling campaigns. It is worth mentioning that Cl showed another dominant peak in the coarse mode during the cold period. The bimodal shape of Cl indicates that it had additional important sources. In our previous study (Angyal et al. 2010), we found that in the coarse fraction, Cl is mainly derived from the winter salting of the streets. This can be confirmed by the fact that there was snowfall the week before the sampling campaign. Except for S, all other elements appeared with smaller contributions at higher MMAD values. In the vegetation period, the mass size distribution of potassium was characterised by a coarse mode with similar MMADs as for Ca and Fe, indicating that the potassium could originate from soil and soil resuspension. In addition, f_C_ also appeared in the coarse mode, with a similar MMAD value. This mode contained 56% of the total f_C_ concentration. It implies that the coarse mode f_C_ definitely represents a non-negligible and important share of atmospheric f_C_ in the city. The main source can be attributed to the bigenic emission, which is high during the vegetation period in Debrecen (Kertész et al. 2024).

Furthermore, in comparison, the content of transition metals (Mn and Cu) was also higher in the coarse mode. These metals are primarily associated with traffic-related coarse particles during warm periods and are usually identified as “road dust” in urban environments (Kertész et al. 2010; Angyal et al. 2011). Moreover, Cu, Zn and Pb could be generated either from brake wear or tyre wear, whereas Mn is mainly related to tyre wear (Sanderson et al. 2014). Besides metals, fossil-derived carbon also showed peaks in the coarse mode in both periods. Glaser et al. (2005) found that a significant amount of black carbon from tyre abrasion and diesel exhaust contributed to highway-influenced soils. Consequently, the fossil-derived carbon peak in the coarse mode is more likely the result of the resuspension of fossil carbon soot particles attached to dust or tyre abrasion.

## Summary and conclusions

In this pilot study, the mass size distribution of atmospheric aerosol pollution, along with the investigation of its elemental components, fossil carbon and contemporary carbon content, was conducted during periods of high and low pollution levels in Debrecen, Hungary, in 2017. By comparing the mass size distribution of carbon with the mass size distributions of trace elements (determined by PIXE), we could acquire new insights into the potential formation, evolution and sources of aerosol particles during both the smog and vegetation periods.

To the best of our knowledge, this is the first study where a detailed mass size distribution of fossil carbon and contemporary carbon has been presented in Europe. The recent developments in the ^14^C analysis with EnvironMICADAS AMS in Debrecen made it possible to measure the two carbonaceous fractions in the size-segregated samples collected by a nano-MOUDI-II type cascade impactor.

The advantage of radiocarbon analysis is that it is the most reliable method for separating the fossil and modern fractions of carbon-emitting aerosols. The uncertainty of the measurement itself is less than 1%. Ten micrograms of carbon in the sample is sufficient for the measurement. This method is well suited for source identification studies.

However, the disadvantage of this method is that it is expensive, and access to measurement is limited. Furthermore, within the modern fraction, it is very difficult to distinguish between biomass burning and biological emissions. In the present study, it was not possible to measure the ultrafine fraction of all components. In order to reliably analyse the ultrafine fraction by both methods, the sampling needs to be improved in the future.

Utilising the results obtained by combining the two methods makes it possible to get information about the sources of PM and their components. The mass size distributions of contemporary carbon and potassium were similar in the whole size range (0.1–10 μm), which unambiguously indicated that a remarkable part of the carbon emission was associated with biomass burning in the smog period. In contrast, the structure of the distribution of potassium for the vegetation period was different from that of contemporary carbon, indicating different sources.

Comparing the mass size distribution of fossil carbon and fingerprint elements, three types of aerosol sources could be distinguished in the droplet mode: fuel-related particles from exhaust emission (tracers: Zn and Pb), coal combustion (tracer: S) and tyre abrasion (tracer: Zn). A relatively high amount of fossil carbon appeared in the coarse mode due to the resuspension of soot particles (from vehicle exhaust) attached to road dust (together with Zn, Pb, Mn and Ni, MMAD > 5 μm).

On the basis of the modal characteristics of tracers, the following sources were also identified: brake abrasion (tracers: Cu and Mn), road dust (Mn, Ni, Cu, Zn and Pb) and mineral dust (tracers: Ca, Ti and Fe) in coarse mode.

The examination of the mass size distribution of carbonaceous fractions, coupled with fingerprint trace elements of urban aerosol pollution, has proven to be a powerful tool for identifying and characterising pollution sources. Building upon this foundation, we plan to conduct further investigations, including the analysis of the elemental (EC) and organic (OC) fractions of carbonaceous aerosol as well.

The main advantage of combining radiocarbon and PIXE analysis in size distribution data is that it can enhance our understanding of the sources of PM and their effects on different size fractions of PM. Compared to other source apportionment approaches (e.g. receptor models) that rely on statistical methods to retrieve source profiles, our approach uses information about the physical characteristics of PM and the radiocarbon content. This allows for source attribution, particularly between fossil and non-fossil sources, with reduced uncertainty. This pilot study can serve as an example of how to utilise these techniques, either as standalone methods or as supportive tools, to achieve better source identification.

### Supplementary Information

Below is the link to the electronic supplementary material.

Supplementary file1 (DOCX 88 KB)

## Data Availability

Data is available upon request.
