# Supplementary material for: Characterisation of urban aerosol size distribution by radiocarbon and PIXE analyses in a middle-European urban environment for source identification: a pilot study
Source: Environ Sci Pollut Res Int. 2024 Jul 11;31(34):47258–74. doi: 10.1007/s11356-024-34215-8 (PMC11297123; doi:10.1007/s11356-024-34215-8)
Supplement: Supplementary file 1 — Supplementary file1 (DOCX 88 KB) [file 11356_2024_34215_MOESM1_ESM.docx]

**Supplementary Material**


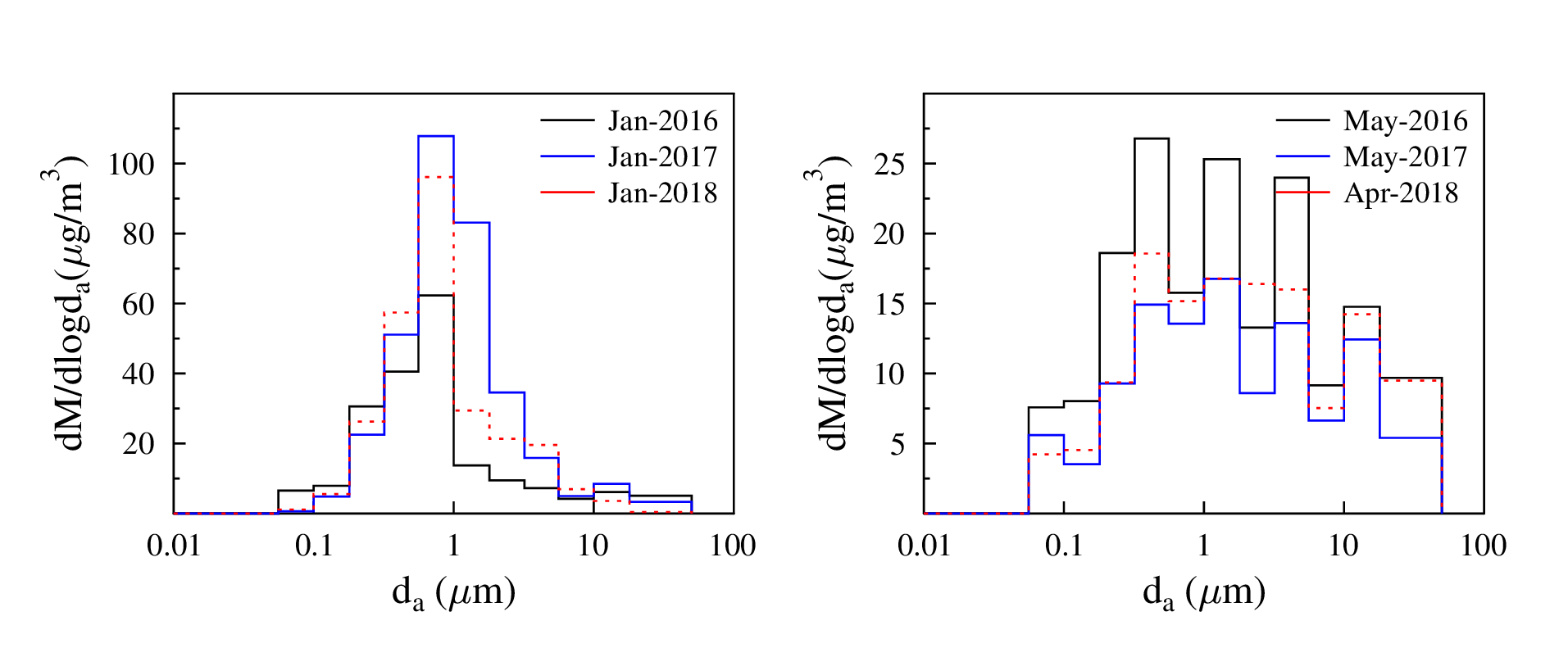


**Figure S1** The size distributions of PM in heating and non-heating periods.

**Table S1**. Pearson correlation coefficients between PM mass concentrations measured at ATOMKI and PM_10_ concentrations measured by Hungarian Air Quality Network stations in 2017.

|  |  | HAQN3 | HAQN2 | HAQN1 | ATOMKI |
| --- | --- | --- | --- | --- | --- |
| HAQN3 |  | 1 | .979(**) | .957(**) | .742(**) |
| N |  | 83 | 83 | 81 | 83 |
| HAQN2 |  | .979(**) | 1 | .950(**) | .722(**) |
| N |  | 83 | 91 | 89 | 91 |
| HAQN1 |  | .957(**) | .950(**) | 1 | .755(**) |
| N |  | 81 | 89 | 89 | 89 |
| ATOMKI |  | .742(**) | .722(**) | .755(**) | 1 |
| N |  | 83 | 91 | 89 | 91 |

**Correlation significant at 0.01 confidence level 2-tailed test

**Table S2.** Pearson correlation coefficients between PM_10_, CO, NO_x_, NO_2,_ and NO concentrations measured by HAQN1.

|  | | PM_10_ | CO | NO_X_ | NO_2_ | NO |
| --- | --- | --- | --- | --- | --- | --- |
| January 15-18 | PM_10_ | 1 | .818(**) | .453(**) | .457(**) | .294(*) |
|  | N | 59 | 59 | 59 | 59 | 59 |
|  | CO | .818(**) | 1 | .628(**) | .523(**) | .624(**) |
|  | N | 59 | 59 | 59 | 59 | 59 |
|  | NO_X_ | .453(**) | .628(**) | 1 | .945(**) | .772(**) |
|  | N | 59 | 59 | 59 | 59 | 59 |
|  | NO_2_ | .457(**) | .523(**) | .945(**) | 1 | .522(**) |
|  | N | 59 | 59 | 59 | 59 | 59 |
|  | NO | .294(*) | .624(**) | .772(**) | .522(**) | 1 |
|  | N | 59 | 59 | 59 | 59 | 59 |
| May 23-25 | PM_10_ | 1 | .489(**) | .503(**) | .505(**) | .325(**) |
|  | N | 73 | 73 | 73 | 73 | 73 |
|  | CO | .489(**) | 1 | .861(**) | .849(**) | .679(**) |
|  | N | 73 | 73 | 73 | 73 | 73 |
|  | NO_X_ | .503(**) | .861(**) | 1 | .996(**) | .711(**) |
|  | N | 73 | 73 | 73 | 73 | 73 |
|  | NO_2_ | .505(**) | .849(**) | .996(**) | 1 | .647(**) |
|  | N | 73 | 73 | 73 | 73 | 73 |
|  | NO | .325(**) | .679(**) | .711(**) | .647(**) | 1 |
|  | N | 73 | 73 | 73 | 73 | 73 |

**Correlation significant at 0.01 confidence level 2-tailed test
